# Pathogenesis of reproductive failure induced by *Trypanosoma vivax* in experimentally infected pregnant ewes

**DOI:** 10.1186/1297-9716-44-1

**Published:** 2013-01-04

**Authors:** Taciana MF Silva, Roberio G Olinda, Carla MF Rodrigues, Antônio CL Câmara, Francisco C Lopes, Wesley AC Coelho, Múcio FB Ribeiro, Carlos IA Freitas, Marta MG Teixeira, Jael S Batista

**Affiliations:** 1 Department of Animal Sciences, Federal Rural University of the Semiarid (UFERSA), Av. Francisco Mota 572, Mossoró, RN, 59625-900, Brazil; 2 Department of Parasitology, Institute of Biological Sciences, University of São Paulo (USP), São Paulo, SP, 05508-900, Brazil; 3 Department of Parasitology, Institute of Biological Sciences, Federal University of Minas Gerais (UFMG), Belo Horizonte, MG, 486, Brazil

## Abstract

The present study was aimed at investigating the effect of experimental infection by *Trypanosoma vivax* in different stages of pregnancy, determining the pathogenesis of reproductive failure, and confirming transplacental transmission. We used 12 pregnant ewes distributed into four experimental groups: G1, was formed by three ewes infected with *T. vivax* in the first third of pregnancy (30 days); G2 comprised three infected ewes in the final third of pregnancy (100 days); G3 and G4 were composed of three non-infected ewes with the same gestational period, respectively*.* Each ewe of G1 and G2 was inoculated with 1.25 × 10^5^ tripomastigotes. Clinical examination, determination of parasitemia, serum biochemistry (albumin, total protein, glucose, cholesterol, and urea), packed cell volume (PCV), serum progesterone, and pathological examination were performed. Placenta, amniotic fluid, blood and tissues from the fetuses and stillbirths were submitted to PCR. Two ewes of G1 (Ewe 1 and 3) presented severe infection and died in the 34^th^ and 35^th^ days post-infection (dpi), respectively; but both fetuses were recovered during necropsy. In G2, Ewe 5 aborted two fetuses on the 130^th^ day (30 dpi) of pregnancy; and Ewe 6 aborted one fetus in the 140^th^ day (40 dpi) of gestation. Ewes 2 and 4 delivered two weak lambs that died five days after birth. Factors possibly involved with the reproductive failure included high parasitemia, fever, low PCV, body score, serum glucose, total protein, cholesterol, and progesterone. Hepatitis, pericarditis, and encephalitis were observed in the aborted fetuses. The presence of *T. vivax* DNA in the placenta, amniotic fluid, blood, and tissues from the fetuses confirms the transplacental transmission of the parasite. Histological lesion in the fetuses and placenta also suggest the involvement of the parasite in the etiopathogenesis of reproductive failure in ewes.

## Introduction

Trypanosomiasis is a disease caused by the pathogenic protozoa of the genus *Trypanosoma*. This parasite has a wide distribution and economic importance in African countries, mainly in regions occupied by its biological vector, the tsetse fly [1]. In West Africa, *Trypanosoma vivax* is considered the most important and pathogenic hemoparasite of livestock promoting reproductive disorders [2,3].

The cyclical transmission through the salivary vector (tsetse fly) is the main mechanism of transmission. In this type of transmission, the parasite completes its entire development cycle in the proboscis of the vector, culminating in the development of metacyclic trypomastigotes, which are infective to the vertebrate host when inoculated with the saliva through the bite of the fly [4]. In contrast, *T. vivax* found in South America is mechanically transmitted only by blood sucking insects of the Tabanidae and Stomoxydae families. These insects act only as carriers and any stage of the parasite cycle of the parasite occurs within the vector. Trypanosomiasis may still be artificially transmitted through the shared use of a needle for several animals during application of medications or vaccinations [5,6]. The adaptation to mechanical transmission has been responsible for the rapid spread and wide geographical distribution of the parasite in areas free of the tsetse fly [7]. Therefore, the presence of *T. vivax* in cattle has already been reported in French Guiana [8], the Atlantic Coast of Colombia [9,10], Bolivia [2,11], and Brazil [12-14].

Despite the evidence of vertical transmission (transplacental) of *T. vivax* in cattle and sheep [15], the precise epidemiological importance of this type of transmission is still unknown. There is speculation that transplacental transmission is associated with the occurrence of abortions, prematurity, intrauterine growth retardation and perinatal mortality [16,17]. Recent studies demonstrate that the trypanosomiasis by *T. vivax* in the semiarid region of Brazil is a debilitating disease, promoting economic losses and serious infection with prevalence reaching 33.8%, 29.7% and 25.4% in cattle, goats and sheep, respectively [18,19]. In a high-mortality outbreak of trypanosomiasis in extensively raised ewes in a non-endemic region in Northeastern Brazil, perinatal mortality due to abortions and neonatal deaths reached nearly 75% [20]. Perinatal mortality was also one of the main manifestations during outbreaks in cattle from other municipalities located in the same region. The authors suggest that transplacental transmission has epidemiological significance for the maintenance and spread of disease in infected herds of ruminants [15].

Despite the evidence of the negative effect of trypanosomiasis caused by *T. vivax* on the reproduction of ruminants, there are still gaps that need to be filled about the pathogenesis of reproductive failure. Thus, the objective of this study was to investigate the effect of an experimental infection with *T. vivax* in pregnant ewes at different stages of pregnancy, to determine the pathogenesis of abortion, and to confirm transplacental transmission by PCR.

## Materials and methods

### Design of the experimental groups

Twelve, approximately 24 month-old pregnant ewes were used. The animals were housed in properly screened individual stalls at the premises of the Center for Studies and Research in Small Ruminants of the Federal Rural University of the Semiarid (UFERSA), Mossoró, Rio Grande do Norte, Brazil.

The study was approved by the Ethics Committee of the Federal Rural University of the Semiarid. Ethical procedures were based on the Brazilian law 6638 (May 8, 1979) “Normas para Prática Didático-Científica da Vivissecção de Animais” and “Ethical Principles for Use of Experimental Animals” from the Brazilian College of Animal Experimentation (COBEA), Brazil, which are in accordance with the “European Convention for the Protection of Vertebrate Animals used for Experimental and Other Scientific Purposes” (Strasbourg, March 18, 1986).

Ultrasonographic exams were performed to identify the gestation period using a 3.5 MHz or 5 MHz abdominal transducer connected to a Logiq Pro 100 GE ultrasound. We estimated the gestational age of the sheep in the initial third using the length of the embryonic vesicle. These animals were infected in the 30^th^ day of pregnancy. The final third of gestation was estimated by the cephalo-coccygeal length of the fetuses, and those ewes were infected in the 100^th^ day of gestation.

Fourteen days before the inoculation of *T. vivax*, the ewes were clinically evaluated and dewormed. Hematological exams were also performed. Then, the animals were allocated into four experimental groups by random selection: Group 1 (G1) was formed by three ewes infected in the first third of pregnancy (Ewes 1, 2, and 3); Group 2 (G2) consisted of three ewes infected in the final third of pregnancy (Ewes 4, 5, and 6); Group 3(G3) was formed by three non-infected ewes with the same gestational period of ewes in G1 corresponding to the control group of this group (Ewes 7, 8, 9); and Group 4 (G4) was composed of three non-infected ewes (Ewes 10, 11, 12) with the same gestational period of ewes in G2 corresponding to the control group of this group. All animals were submitted to identical management conditions, fed with water and Tifton (*Cynodon* sp.) hay *ad libitum*, and supplemented with commercial concentrate (1.5% BW/day/ewe).

### Inoculum preparation and experimental infection with *T. vivax*

The strain of *T. vivax* used for experimental infection of the ewes in this study was derived from a natural outbreak in ewes from the city of São João do Rio do Peixe, Paraíba, Northeastern Brazil [20]. Blood samples were collected from ewes with parasitemia in 10% ethylenediaminetetraacetic acid disodium (EDTA) mixed with 8% glycerol, and frozen in liquid nitrogen (−196°C). Immediately before inoculation, the strain sample was defrosted at room temperature. Each ewe from the experimental group was inoculated intravenously with 1 mL of blood containing 1.25 × 10^5^ trypomastigotes of *T. vivax*, which was estimated according to Batista et al. [18].

### Determination of parasitemia and hematocrit

Parasitemia was determined daily by research of the trypanosome in the blood collected from small blood vessels located in the ear, using a blood smear between the slide and coverslip, according to the technique described by Batista et al. [18]. The evaluation of hematocrit was performed daily by microcentrifugation.

### Clinical examination of the ewes and weight of the newborns and aborted fetuses

Experimental ewes were examined daily to assess rectal temperature, status of the mucous membrane, and external lymph nodes, and were checked for signs of labor or abortion. Body score was determined weekly using a scale of 0 (for very thin animals) to 5 (for fat animals) [21]. At the beginning of the experiment, all ewes were classified with the help of body condition scores ranging from 3.5 to 4. Immediately after delivery or abortion, newborns and fetuses were measured and weighed with an electronic digital scale.

### Serum biochemistry and analysis of progesterone

In weekly intervals, blood was obtained by jugular vein puncture, and placed in sterile tubes containing 10% EDTA for the determination of serum biochemistry. Albumin, total protein, glucose, cholesterol, and urea were determined using commercial kits (Katal, Belo Horizonte, MG, Brazil) and an automatic analyzer SBA-2000 (Celm, Barueri, SP, Brazil). A blood sample was also placed in sterile Vacutainer tubes for determination of plasma progesterone by microparticle enzyme immunoassay (Immulite 2000, PRG, Progesterona, Siemens Healthcare Diagnostics Products Limited).

### Pathological and histopathological studies

Pathological examination of the aborted fetuses, stillborns, placenta, and umbilical cords was performed. Fragments from thoracic and abdominal organs, and the central nervous system from the fetuses and stillborns were fixed in 10% buffered formalin and then embedded in paraffin. Sections of 5.0 μm were cut using a microtome, and stained using the classical hematoxylin–eosin method (HE).

### Diagnosis of *T. vivax* by PCR

Samples from amniotic fluid (1 mL) and fragments of approximately 1 cm^3^ of each placenta, stillborn and aborted fetus organs were collected, and preserved in 99% ethanol. The DNA preparations were subjected to a highly sensitive PCR assay specific for *T. vivax* standardized by Cortez et al. [22]. This PCR method targets repeated gene sequences that encode cysteine proteases (Cathepsin L) and was carried out using the oligonucleotides Tvi2 (forward: 5’ GCC ATC GCC AAG TAC CTC GCC GA 3’) and DTO156 (reverse: 5’ TTAGAATTCCCAGGAGTTCTTGATGATCCAGTA 3’) as primers. The diagnosis was confirmed by PCR and amplifying a DNA fragment of about 177 base pairs (bp) that is also observed in the DNA of *T. vivax* (from Catolé do Rocha, Paraiba, Northeastern Brazil), which was used as a positive control. DNA samples from amniotic fluid, blood, and tissues of the non-infected ewes (G3 and G4) were used as negative controls.

### Statistical analysis

Data were expressed as mean ± standard error, and analyzed by SAS statistical software (SAS Institute Inc., Cary, North Carolina, USA) version 9.0, and Minitab (Minitab Inc. LEAD Technologies), version 16.1.1. Primarily, the data were assessed for normality by Lilliefors (Kolmogorov-Smirnov) and homogeneity by Levene. Statistical difference between the values of the infected groups G1 and G2, with their respective controls (G3 and G4) were calculated by the independent t test when showing normality, and by Mann- Whitney test when not-normal. *P* < 0.05 was considered significant. In G1 and G3, statistical analysis comprised until the 34^th^ day post-infection (dpi) (64 days of pregnancy) period in which all the ewes in G1 were still alive.

## Results

### Mean parasitemia

The period after inoculation of *T. vivax* and the onset of parasitemia (pre-patent period) was three days, and remained high throughout the experimental period, and achieved the peak of parasitemia on the 43^rd^ (13 dpi) and 115^th^ (15 dpi) days of gestation in G1 and G2, respectively.

### Clinical signs and reproductive changes

**Figure 1:**
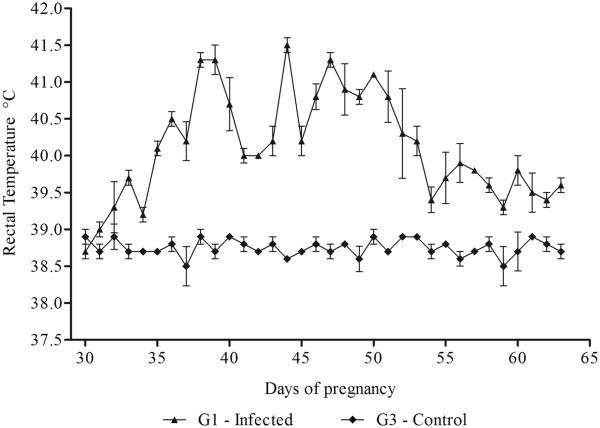
**Evolution of the rectal temperature of sheep after inoculation with *T. vivax *and negative control.** Mean values of rectal temperature (°C) in ewes experimentally infected with *T. vivax* in the first trimester of pregnancy (G1) and the control group (G3).

**Figure 2:**
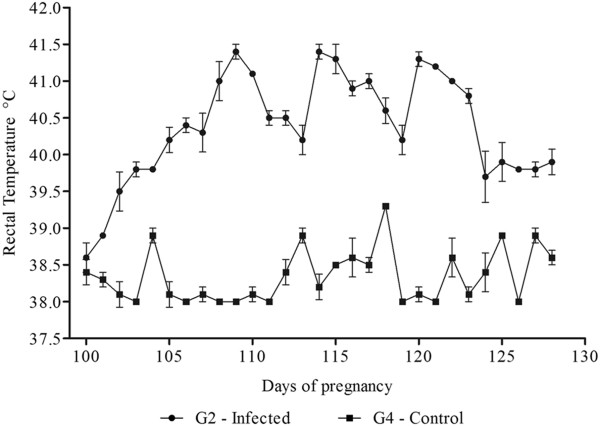
**Evolution of the rectal temperature of sheep after inoculation with *T. vivax *and negative control.** Mean values of rectal temperature (°C) in ewes experimentally infected with *T. vivax* in the final trimester of pregnancy (G2) and the control group (G4).

Hyperthermia was observed in G1 between 35 (5 dpi) and 53 (23 dpi) days of pregnancy with a significant difference (*P* < 0.05) compared to G3. In G2, hyperthermia was significantly different (*P* < 0.05) from G4 between 105 (5 dpi) and 123 (23 dpi) days of gestation (Figures 1 and 2). Clinical signs included apathy, tachypnea, tachycardia, hyporexia, pale mucosae, and enlarged lymph nodes. Ewes 2 and 3 presented severe diarrhea between the 37^th^ (7 dpi) and 48^th^ (18 dpi) days of pregnancy. Ewes from both infected groups showed progressive weight loss. Ewes 1 and 3 presented a worsening of symptoms and showed prostration, weakness, lethargy, severe anemia (PCV minimum of 8.0%), anorexia, and recumbence; dying in the 64^th^ and 65^th^ days of pregnancy (34 and 35 dpi), respectively. These ewes presented body condition score of 1.5, whilst Ewe 2 presented a body score of 2. G2 (Ewes 4, 5 and 6) presented body condition score of 2 in the final days of the experiment. Ewes from G3 and G4 maintained the body score observed at the beginning of the trial.

Fetuses from the dead ewes (Ewe 1 and 3) were recovered during necropsy. The fetus from Ewe 1 was 7 cm in length and weighed 0.5 kg; the fetus from Ewe 3 was the same length and 0.6 kg. Ewes 2 and 4 delivered two weak lambs weighing 1.5 and 1.1 kg, and measuring 23 and 21 cm, respectively. These ewes had a poorly developed udder and agalactia. So the lambs were not able to ingest colostrum, and died five days after birth. Approximately, on the 130^th^ day (30 dpi) of pregnancy, Ewe 5 aborted two lambs that were nearly 25 cm in length and weighed 1.2 and 1.3 kg. Ewe 6 also aborted a 23 cm and 1.5 kg fetus in the 140^th^ day (40 dpi) of gestation.

### Packed cell volume, serum biochemistry and analysis of progesterone

Significant reduction (*P* < 0.05) in the mean PCV was observed in G1 and G2 compared to G3 and G4, respectively. The average PCV in the G3 and G4 remained within the normal parameters for the species during the whole experimental period. Our data showed that mean values of serum glucose, total protein, albumin and cholesterol decreased significantly (*P* < 0.05) in G1 compared with G3. Serum albumin level was the only serum parameter that decreased significantly (*P* < 0.05) in G2 compared with G4. There was a gradual increase in serum urea and a significant difference in mean values of G1 and G2, when compared to their respective control. In G2, a marked reduction in progesterone levels and a significant difference compared to G3 (*P* < 0.05) was also observed (Table 1).

**Table 1:** Mean values ± standard error for the infected and control groups according to the variables studied

**Variables**	**Period**	**Infected**	**Control**	**P value**
Glucose (mg/dl)	G1	38.0 ± 7.71	71.42 ± 1.50	0.008*
	G2	49.25 ± 9.62	70.5 ± 2.10	0.14
Total protein (g/dl)	G1	5.15 ± 0.53	7.07 ± 0.08	0.02*
	G2	5.67 ± 0.75	7.12 ± 0.13	0.148
Albumin (g/dl)	G1	2.30 ± 0.19	3.51 ± 0.03	0.0017*
	G2	2.62 ± 0.38	3.52 ± 0.04	0.03*
Cholesterol(mg/dl)	G1	54.28 ± 5.26	72.42 ± 1.08	0.015*
	G2	61.5 ± 9.64	74.25 ± 0.94	0.24
Urea (mg-dl)	G1	53.42 ± 9.84	14.42 ± 0.94	0.01*
	G2	44.25 ± 13.3	13.5 ± 1.55	0.02*
Progesterone (ng/dl)	G1	12.99 ± 1.97	17.4 ± 0.69	0.10
	G2	9.56 ± 2.80	18.63 ± 0.29	0.007*

* Meaningful statistical difference (*P* < 0.05).

### Pathological and histological studies

Gross findings included enlarged, hemorrhagic, friable cotyledons and focal areas of necrosis in the placentas of Ewes 5 and 6. All aborted fetuses and stillbirths showed a moderate amount of sero-bloody fluid in the abdominal and thoracic cavity, and pericardial sac. Subcutaneous edema and congestion of the liver, kidneys, lungs, and brain were also noted. At the heart of one of the aborted fetuses, petechial hemorrhages and suffusions in the epicardium were observed.

**Figure 3:**
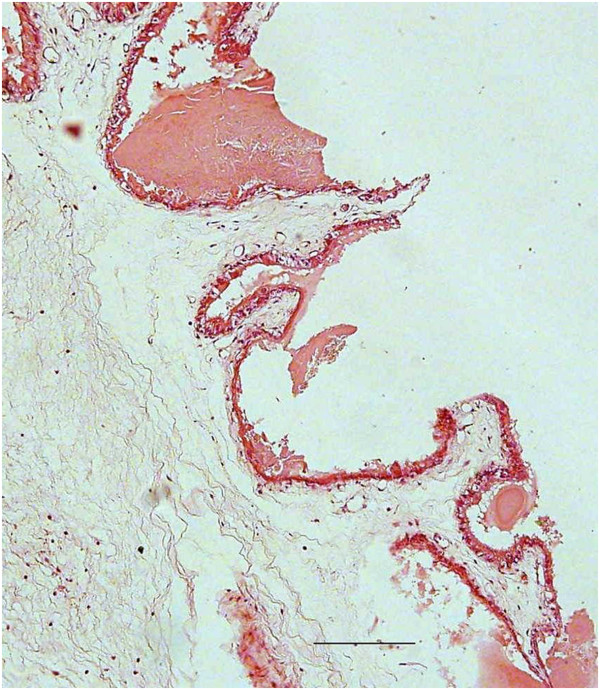
**Placental injury induced by the presence of *T. vivax *in sheep.** Necrosis of the chorionic epithelium of the placenta of ewes experimentally infected with *T. vivax* in the final trimester of pregnancy. Hematoxilin-eosin, obj. 10×, Scale bar = 150 μm.

**Figure 4:**
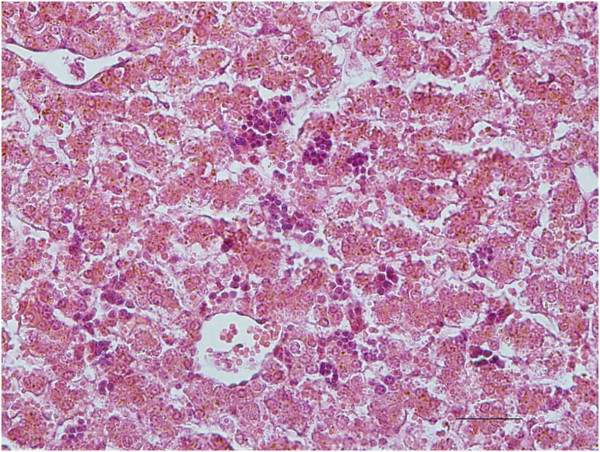
**Inflammatory processes in the liver parenchyma of fetuses induced by the migration of *T. vivax.*** Diffuse hepatocellular necrosis and multifocal mononuclear hepatitis in a fetus aborted in the final trimester of pregnancy*.* Hematoxilin-eosin, obj. 40×, Scale bar = 99 μm.

**Figure 5:**
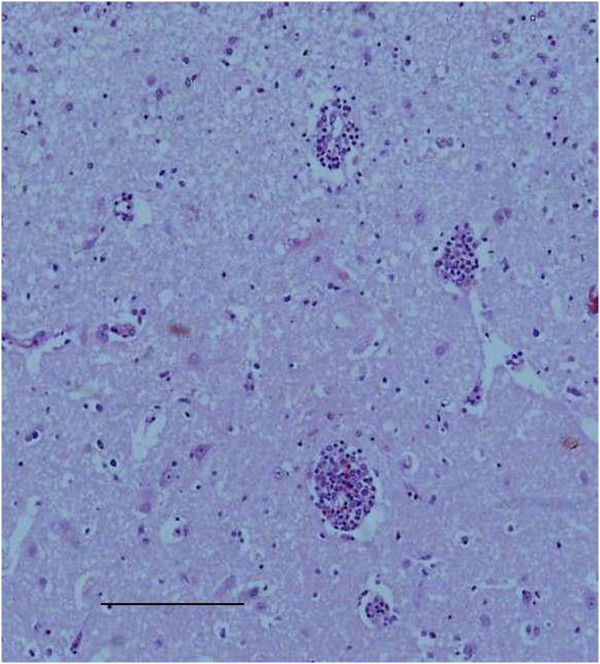
**Inflammatory processes in the brain of fetuses induced by the migration of *T. vivax. *** Mononuclear multifocal encephalitis in a fetus aborted in the final trimester of pregnancy. Hematoxilin-eosin, obj. 10×, Scale bar = 147 μm.

Histologically, necrosis of the chorionic epithelium of the placentas of Ewes 5 and 6 was observed (Figure 3). In the liver of the aborted fetuses, hepatitis was characterized by multifocal lymphocytic inflammatory infiltrates, and diffuse hepatocellular necrosis (Figure 4). The presence of extensive pericarditis characterized by the inflammatory infiltration of lymphocytes and plasma cells; and multifocal lymphocytic encephalitis located in the white matter of the brain was also noted (Figure 5).

### Detection of parasite DNA in fetal tissue and placenta

**Figure 6:**
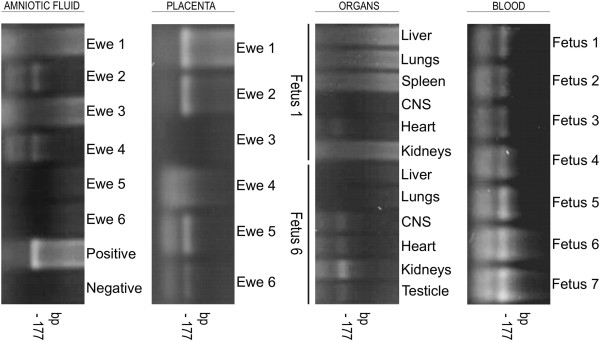
**Polymerase chain reaction illustrative of blood samples and tissue.** Agarose gel stained with ethidium bromide showing results from PCR reactions targeting Cathepsin L-like gene of *T. vivax* (fragment of ~177pb) in the amniotic fluid and placenta from experimentally infected ewes; blood and tissues from the fetuses.

PCR analysis for *T. vivax* (TviCatL-PCR) showed the amplification of a DNA fragment of approximately 177pb, specific of *T. vivax,* and retrieved from the cathalitic domain of the Catepsina L gene (visualized in 2% agarose gel). The samples resulted positive in the blood of the fetuses (100%), placentas (57.1%), amniotic fluid (28.6%), heart (28.6%), nervous system (14.3%), kidneys (14.3%) and testicles (14.3%) of the fetuses. However, PCR was negative for all samples of the control group (Figure 6).

## Discussion

Our results indicate that pregnancy changed the clinical aspects of trypanosomiasis, since there was an exacerbation of the infection, manifested by high and persistent parasitemia followed by hyperthermia. Studies evaluating follicular degeneration in goats experimentally infected with *T. vivax,* using the same dose (1.25 × 10^5^ trypomastigotes) and inoculum from the present study, showed high parasitemia and hyperthermia in the acute phase only, whereas in the chronic phase, parasitemia is either absent or of low intensity. An important feature is that none of the experimental animals died [23].

Mean values of hematocrit (PCV) showed the typical evolution of trypanosomiasis, with a progressive reduction in the values throughout the infection period. Evaluation of the degree of anemia by hematocrit determination has been frequently used in monitoring the evolution of the disease, since this hematological parameter is most frequently changed in animals experimentally or naturally infected by *T. vivax*[24-26]. The hematocrit showed a dramatic decrease in infected ewes from this study, reaching 8%. Low hematocrit is associated with more severe stages of trypanosomiasis and may have contributed to the fatal course of disease in Ewes 1 and 3; and to the reduced body weight of newborn lambs and aborted fetuses.

Pathological consequences of the infection in pregnant females appear to be directly related to the gestational period in which it occurs. In the present study, two ewes infected in the first third of pregnancy presented serious infection and died within 65 days of pregnancy (35 dpi). However, the mortality of the ewes infected in the final third of pregnancy did not occur. Two of those ewes aborted, and another delivered a live lamb; but the weak offspring died five days after birth. The negative effect of trypanosomiasis in pregnant females is probably a consequence of the low immunity in this period associated to hematological and biochemical alterations promoted by the hemoparasite, that also increases the metabolic needs of the pregnant female [27-29]. In addition, the ovine placenta starts secreting progesterone around day 55, which is sufficient to maintain pregnancy in most ewes when the corpus luteum is removed [30]. Therefore, the damage to the placenta caused by the protozoa may promote the insufficient placental secretion of progesterone and the consequent interruption of pregnancy.

The evaluation of blood glucose concentration has been applied as an indicator of energy metabolic activity. Thereby, the metabolism of pregnant ewes is characterized by high glucose requirements [31]. The effect of *T. vivax* on the energetic metabolism in goats promoted an increase of 25% in the energy requirements for maintenance. Hypoglycemia is a common finding in acute trypanosomiasis, and is attributed to energy expenditure caused by hyperthermia and blood glucose consumption by trypanosomes [32]. In this study, hypoglycemia revealed to be an important biochemical change observed in the infected groups. Thus, the combination of increased energy demands in pregnancy associated with a negative energy balance promoted by the infection is incompatible with the maintenance of fetal development in pregnant ewes. In addition, fetal fructose is produced by the placenta from glucose, and comprises about 70-80% of fetal blood sugar. The values of fetal fructose are correlated with the maternal glucose levels, and the maintenance of high concentrations can be seen as an indicator of ideal placental function [33]. Another important metabolic alteration in the infection is the increase in protein catabolism, which is evidenced by the significant decrease of serum total protein and increased urea [34]. A similar trend was observed in this study, in which low serum levels of total protein and high levels of urea were observed in the infected ewes.

Another systemic alteration observed in infected pregnant ewes was the progressive loss of body condition, which was manifested by low body scores achieving the lower limit of 1.5 (Ewe 1 and 3). Whatever the cause, small ruminants with a body condition of 1.5 are nine times more prone to abort compared with those in good body condition [35]. Other systemic manifestations observed in this group, and found in the reviewed literature as capable of promoting abortion, are hyperthermia and anemia [36,37]. The reduction in serum progesterone is another cause of maternal origin that can trigger abortion. In this study, it was observed that the serum levels of progesterone of infected ewes were significantly lower than those observed in the control group. It is known that the decrease in progesterone may result in disruption of pregnancy and promotes the expulsion of the dead fetus. In female animals, hormonal disorders in trypanosomiasis caused by *T. vivax* occur due to degeneration of the hypothalamus, pituitary, and gonads; which results in disruption of hormonal secretion, and consequent diminishing plasma concentrations of those hormones, which are fundamental for the reproductive processes, including pregnancy [38]. Another hypothesis for the abortions consists in the occurrence of maternal hypoglycemia and, consequently, fetal hypoglycemia. This event is followed by the successive events of an increase of corticosteroids, estrogen, and prostaglandin, causing luteolysis and abortion [39].

The demonstration of parasite DNA by PCR in the placenta, blood, and tissue from aborted fetuses and stillbirths suggest that the protozoan traverses the maternal bloodstream. Thus, the infection invades the pregnant uterus, causing placental damage, and spreads into the blood and fetal tissues. Our histopathological results showed pericarditis, hepatitis, and encephalitis in the fetuses, therefore classifying these abortions as of infectious origin [40].

The abortion or birth of weak lambs may also be explained by placental insufficiency. Reproductive failure due to chorionic epithelial damage may occur due to inadequate nutrition or fetal oxygenation. In such cases, the fetus suffers anoxia, releasing the adrenocorticotrophic hormone with the subsequent release of fetal cortisol, which stimulates the production of estrogen and prostaglandin F2α by the placenta. This event results in luteolysis, with a consequent decrease in progesterone [41]. The presence of the DNA of *T. vivax* in the placenta associated with its damage suggests that the parasite has an important role in the pathogenesis of placental dysfunction and abortion.

The detection of *T. vivax* in the placenta, amniotic fluid, fetal blood, and tissues is an unprecedented event, and confirms the first diagnosis of the transplacental transmission through detection of the parasite DNA by PCR in ewes. Although little investigated, transplacental transmission was confirmed for the first time in 1972 by the finding of large numbers of trypanosomes in the blood four hours after the birth of lambs from experimentally inoculated ewes with *T. vivax* in the final trimester of gestation [16]. Later, there was the confirmation of the transplacental transmission of *T. vivax* in cattle by the detection of high parasitemia in a calf born from an infected cow in the same period of pregnancy [42]. In Venezuela, congenital transmission was also observed in a calf, in which parasitemia and anti-*T. vivax* antibodies were detected by indirect immunofluorescence [43]. Recently, in Brazil, the probable transplacental transmission was recognized in four three-day-old calves with high parasitemia and born from chronically infected cows [15]. These authors attribute the high prevalence of trypanosomiasis by *T. vivax* in Northeastern Brazil to the transplacental transmission, as the regional climate (hot and dry) does not favor the development of host insects during most of the year.

In naturally infected flocks and herds, the transplacental transmission of the parasite is not well known and is underestimated by farmers and practitioners. The published cases of placental transmission by *T. vivax* do not describe lesions and parasitism of the placenta, and show no morphological evidence of the infection in the fetus. In this study, the occurrence of transplacental transmission in ewes was based on the identification of pathological lesions, suggesting protozoan infection, and also by the detection of the parasite’s DNA in the placenta, amniotic fluid, blood, and tissues from aborted fetuses and recently dead neonates. The transplacental transmission of *T. vivax* is unquestionable, as well as its effects on fetuses and newborns, which may occur in the initial or final third of gestation. It is likely that this type of transmission contributes to the survival of the parasite and the spread of infection in herds, and is also associated with cases of abortion, premature births, low birth weight, and perinatal mortality. These facts are often reported during the outbreaks of *T. vivax* infection in the Brazilian semiarid region [15,18,20].

Our experiment confirms the importance of *T. vivax* as a causative agent of abortion and perinatal mortality, as previously demonstrated in the outbreaks of infection in cattle, ewes, and goats in the Brazilian semiarid region [14,19,20,44]. Our data suggest that *T. vivax* is able to cause abortions or perinatal mortality in different ways. In fact, the reproductive disturbances caused by the parasite may be multifactorial, since maternal systemic effects and injury of the placenta and fetus were observed.

## Abbreviations

PCR: Polymerase chain reaction; PCV: Packed cell volume; DNA: Deoxyribonucleic acid; UFERSA: Federal Rural University of the Semiarid; EDTA: Ethylenediaminetetraacetic acid disodium; HE: Hematoxylin–eosin method; Dpi: Days post-infection.

## Competing interests

The authors declare that they have no competing interests.

## Authors’ contributions

JSB carried out the pathological analyses, conceived the study, and participated in its design and coordination. TMFS, RGO, ACLC, FCL, MFBR and CIAF carried out the experimental infection, clinical exams, and drafted the manuscript. MMGT and CMFR carried out the DNA analysis. JSB, WACC and TMFS performed the statistical analysis. All authors read and approved the final manuscript.
